# Teacher voices matter: The role of teacher autonomy in enhancing job satisfaction and mitigating burnout

**DOI:** 10.1371/journal.pone.0317471

**Published:** 2025-01-13

**Authors:** Cheyeon Ha, Tim Pressley, David T. Marshall

**Affiliations:** 1 The Child Study Center, Yale School of Medicine, Yale University, New Haven, Connecticut, United States of America; 2 Department of Psychology, Christopher Newport University, Newport News, Virginia, United States of America; 3 College of Education, Auburn University, Auburn, Alabama, United States of America; Universidad de Zaragoza, SPAIN

## Abstract

This study aims to explore variables associated with teacher burnout at the end of the 2021–2022 school year and shed light on teachers’ mental health issues related to psychological factors. We collected survey data from 824 United States teachers. Using a moderated mediation analytic model, the results of this study showed that teachers’ autonomy had considerable interaction effects on job satisfaction and burnout with other variables in this study (i.e., administrative support on job satisfaction and job satisfaction on burnout). Teachers with more experience and higher job satisfaction showed lower burnout scores. Furthermore, results showed significant relationships between key variables influencing burnout. These findings are key as teacher attrition grows across the United States, and school leaders should look to support teachers’ well-being.

## Introduction

Teachers have faced many challenges and changes to traditional teaching approaches during and after the COVID-19 era [1,2]. Since 2020, schools have asked teachers to move instruction virtually, teach in-person and virtual students simultaneously, and learn new instructional techniques, all while implementing COVID-19 safety protocols. Research conducted early in the pandemic found that teachers had high burnout and anxiety levels and felt overworked [3,4]. Schools reopened in the fall of 2020 using various learning modalities that differed across contexts [5–7]. During the first month of the 2020–2021 school year, teachers reported high levels of burnout associated with COVID-19 anxiety, administrative support, and communication with parents [3]. Though some teachers had decreased anxieties during the first month of returning to providing instruction in fall 2020, most teachers maintained or saw an increase in anxiety, especially teachers who taught virtually or had high levels of burnout entering the school year [2,3,8].

As the 2020–2021 school year progressed, teachers adjusted instruction based on COVID-19 metrics and instructional demands. Teachers continued to feel high levels of stress, and burnout due to the new requirements of the instructional approaches asked of them, the limited support received through the continual changes, and the increase in teacher workloads [e.g., 4,9]. In addition, teachers reported increased workloads and extended teaching hours as society continued to work through the pandemic [10]. With the increased workloads and demands on teachers, researchers, and school leaders worldwide have found an increase in teacher burnout [11–13]. This increased workload caused school leaders to be concerned about increased teacher attrition [11,14]. Without the required resources, the likelihood of burnout would only increase. In addition, Bryner [15] pointed out the issue of teacher mental health and burnout related to the recent teacher shortage in K-12 education in the United States. Consequently, it is important to understand teachers’ mental health and job satisfaction changes in the post-COVID-19 teaching environment to provide helpful suggestions for all stakeholders in K-12 education.

**Fig 1:**
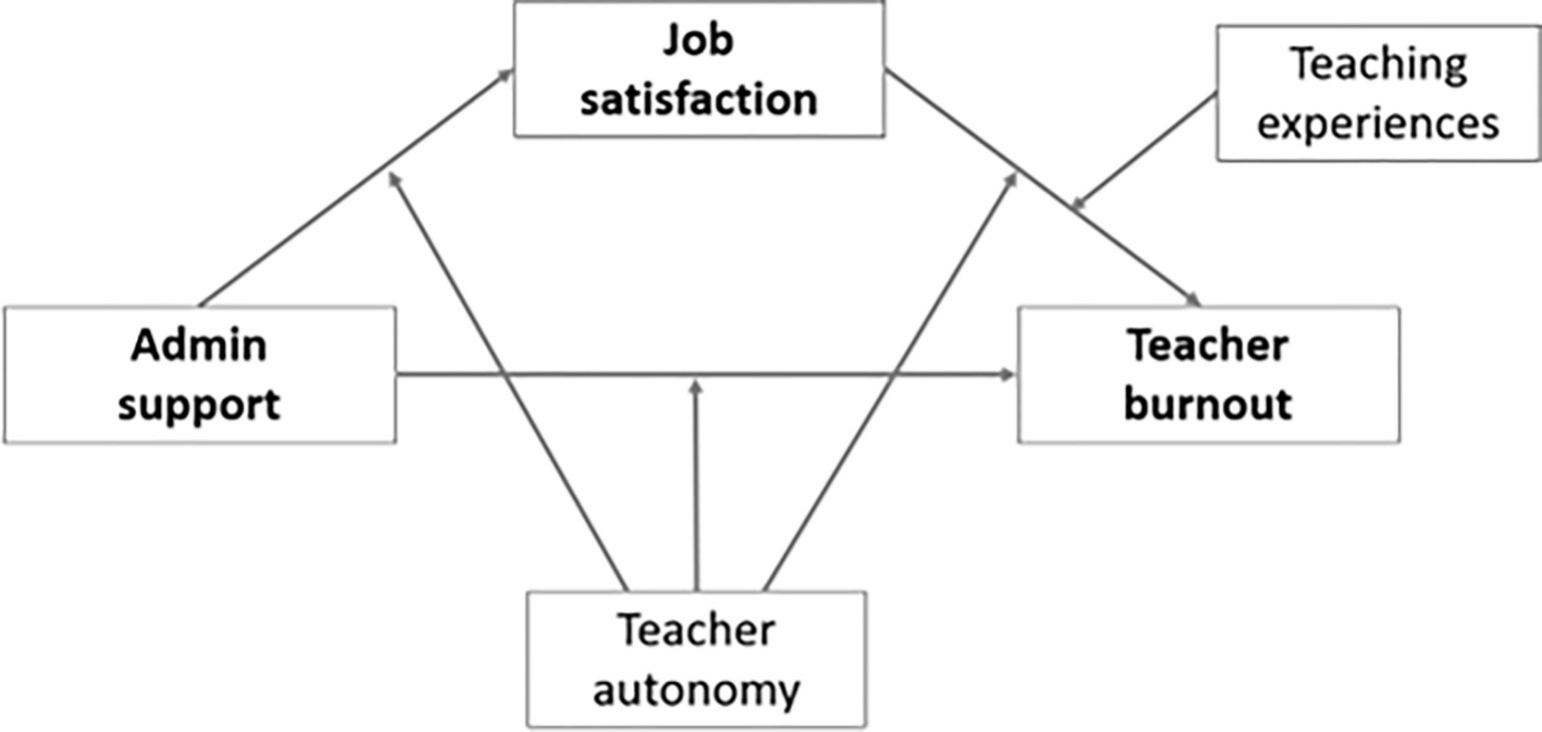
A conceptual moderated mediation model of this study.

For this study, we reviewed multiple recent studies that have explored teachers’ stress and burnout topics. Previous empirical evidence supports a link between school administrative support to higher job satisfaction [16,17] and lower teachers’ burnout [3,18]. Also, multiple theoretical studies have explained that perceived positive autonomy is critical to people’s psychological well-being [19,20]. Based on the previous findings, we suggested a conceptual model of teachers’ burnout related to teaching during a pandemic (see Fig 1). In this model, we assumed meaningful path regressions among administrative support, job satisfaction, and teachers’ burnout. We additionally tested the possible moderating effects of teachers’ perceived autonomy on the path regression in our conceptual model.

## Theoretical framework

The Jobs Demands-Resources (JD-R) model focuses on two main factors in a career (i.e., job demands and job resources) that are associated with stress [21]. These two factors may affect a person’s burnout level depending on the demands and resources in the job setting. According to the model, job demands make up required mental, physical, and emotional tasks that come with costs, while job resources are factors that support a person in (1) reaching a work goal, (2) reducing the required job demand costs; and/or supporting personal growth [21]. In order to support a person’s well-being at work, there must be an appropriate balance between job demands and resources. Due to the nature of the framework and the needs and requirements of the teaching professions, the jobs-demand resource model fits well within the teaching context. Job demands may include lesson planning, grading, and emotional demands, while job resources may include administrative support, available resources to support teachers and learning, professional development, and teacher autonomy [22].

Throughout the COVID-19 pandemic, the demands only increased for most teachers, and the resources needed changed due to the ever-changing context of teaching during the pandemic [23]. A study of Austrian early childhood educators using the JD-R model [24] used latent profile analysis and found three groups of teachers. Teachers who either had moderate demands and high resources or high demands and moderate resources fared better than other teachers. Teachers with high demands and low resources reported feeling more exhausted and less engaged in their work than peers with more resources. Teachers faced new demands during the pandemic and required new resources to do their jobs. Some of the new demands included learning new learning management systems, adapting instruction to support HyFlex and virtual learning [25–27], health demands, including the fear of catching and spreading the COVID-19 virus [27,28] and implementing a range of mitigation strategies in school to curb viral spread [29]. To support these new demands, teachers needed new resources during the pandemic, such as proper administrative support for new learning approaches [3,9,30], support for teacher self-efficacy [31], and overall teacher well-being [32]. Since schools began to return to normal by the end of the 2021–2022 school year, it is important to understand the demands and resources that may influence teacher burnout and well-being.

The current study was developed based on the model explored by Pressley, Ha (3) of teachers in the United States during the fall of 2020. The model found that less administrative support had a negative relationship with teacher engagement efficacy and a positive relationship with teacher stress. In addition, low instructional efficacy and high anxiety were related to higher teacher stress. Furthermore, Pressley, Ha (3) found a significant indirect effect of teacher efficacy between administrative support and teacher stress. These results aligned with similar findings collected early in the pandemic from Europe and Canada [9,31].

### Teacher burnout

Even before the pandemic, teacher burnout was an issue for schools. Previous studies have found that day-to-day work requirements and busy days lead to higher rates of burnout for teachers [33,34]. Specifically, teachers felt overworked due to implementing new curriculums and initiatives, hectic workdays, and having limited time to complete tasks such as preparing for teaching, making necessary copies, and communicating with stakeholders [33,34]. In addition, the heavy workload led teachers to exhaustion, lower self-efficacy, and lower self-esteem [34,35]. One key contributor to teacher burnout within the school environment before and during the pandemic is school administrators’ support [e.g., 3,9,36,37]. During the pandemic, administrators played a critical role in how teachers viewed burnout, with more burnout teachers blaming school administrators for the issues that led to higher levels of burnout [9,13].

In a national survey in the United States conducted in the spring of 2022, teachers reported all-time low job satisfaction scores [38]. Reasons teachers considered leaving the classroom included an increased workload, with teachers reporting working 54 hours a week on average and the need to support kids academically and socially; in addition, teachers did not report feeling respected, and 44% of teachers shared that they planned to leave teaching in the next two years [38]. Similarly, Marshall, Pressley [39] found that three-fourths of teachers thought about leaving during the 2021–2022 school year, with over half exploring non-teaching jobs and a quarter applying for non-teaching jobs.

Throughout the pandemic, teachers experienced burnout at a high rate due to the new challenges and expectations put on teachers [40,41]. Reports of teacher burnout have varied across studies during the pandemic, as Agyapong, Obuobi-Donkor [40] found that 25–74% of teachers reported feeling burnout in a meta-analysis of 70 studies. In another previous study, Kotowski, Davis [42] found results falling within the same range, with 57% of teachers reporting very or extreme burnout in the spring of 2021. With such a staggering number of teachers feeling burnout, it is important to understand factors that continue to contribute to teacher burnout as society works back to normalcy.

### Teacher well-being and feeling autonomy

One factor that has impacted teachers during the COVID-19 pandemic is teachers’ mental health and well-being, with many teachers reporting increased stress, anxiety, and depression [e.g., 32,43,44]. Kim, Oxley [44] attributed these mental health issues to a variety of factors, including additional job demands (e.g., increased workload, multiple roles, and uncertainty) and a lack of job resources (e.g., social support, work autonomy, and coping strategies). Teachers also reported high stress levels, burnout, and job dissatisfaction due to the challenges they faced with online teaching during COVID-19 [32,41,45]. Other stressors included having concern for student well-being and being frustrated with the level of support they received from school administration [23]. One final factor that influenced teacher well-being was increased anxiety and distress. Hutchison, Watts [28] found that during the second year of the pandemic, teachers reported higher anxiety levels and distress than before the pandemic began.

The decrease in teacher well-being has not been limited to just the United States, as Billaudeau, Alexander (43) found factors such as teacher autonomy and feeling safe at school factors impacting teacher well-being in France, Canada, Belgium, Gambia, and Morocco. In addition, Billaudeau, Alexander [43] found that Australian teachers reported high levels of stress and lower levels of joy and positivity during the COVID-19 pandemic, which negatively impacted teacher well-being. Ample psychological research highlights that experiencing higher autonomy is vital to psychological well-being [19,46]. Perceived autonomy is linked to positive emotions (e.g., job satisfaction) [47] and higher engagement levels in the workplace [46]. In the empirical study on teachers’ well-being, Ebersold, Rahm [48] found that teachers’ perception of autonomy support by the principal was related to their lower negative and higher positive affect. It was also linked to lower emotional exhaustion. These findings align with the idea that affording teachers more autonomy in the classroom can contribute to teachers’ positive job experiences; thus, it may reduce teachers’ burnout.

This study focused on the teachers’ autonomy and explored its interaction with other teacher-related variables. Previous research pointed out that teachers’ feeling of autonomy is deeply related to their mental health and well-being [16]; it may also contribute to students’ well-being and academic excellence [49]. For example, teachers’ feeling of autonomy was strongly related to their perceived professionalism [16]. Also, Marshik, Ashton [49] reported that teachers’ perceived autonomy at schools impacts their students’ procedural and cognitive autonomy; for example, elementary students working with teachers who had higher feelings of autonomy showed better reading achievement. Efforts to recover teacher autonomy will be necessary to improve teachers’ well-being and help students return to normal in the post-COVID-19 era.

## Purpose of this study

This study aims to explore variables associated with teacher burnout at the end of the 2021–2022 school year and shed light on the teachers’ mental health issues in recent years. As teachers returned to a more normal year in 2021–2022, teachers faced lofty expectations that included working to eliminate student learning loss from the pandemic [e.g., 50], supporting student behavior and mental health within the classroom, and providing rigorous instruction to cover current grade level and subject standards. Therefore, we explored the relationship between administrative support, job satisfaction, and teacher burnout, exploring whether the relationship between the three variables might show different patterns depending on the teachers’ perceived autonomy level. This study also investigated whether teaching experience has different moderation effects between teachers’ job satisfaction and burnout. Fig 1 shows a conceptual model of this study, and we answer the following research questions (RQs) by exploring the moderated mediation model.

1. What relationship exists among administrative support, teachers’ job satisfaction, burnout, perceived autonomy, and teaching experiences?
2. Is the relationship between administrative support and teacher burnout mediated by job satisfaction?
3. To what extent is administrative support related to job satisfaction and teacher burnout, and does teacher autonomy moderate these relationships?
4. To what extent is job satisfaction related to teacher burnout, and does teaching experience moderate this relationship?

## Methods

### Study design and procedures

Following a cross-sectional study design, this study collected the survey data from 824 in-service teachers in the United States during the post-COVID era (i.e., 2021–2022 academic year). The study explored the mechanism of teachers’ burnout with job satisfaction, perceived administrative support, and feeling of autonomy at work. We tested a priori power test for a multiple regression model with seven predictors; the required sample sizes were 103 with medium effect size and 721 with small effect size to have the desired statistical power .80 level.

Following the data collection guidelines of the Institutional Review Board (IRB), we collected data using the online surveys from May 2022, until obtaining a sufficient sample size at the desired statistical power level. To be eligible to participate in the research, individuals had to be currently employed as a PreKindergarten-12^th^ grade (PK-12) teacher in the United States at the time of the survey administration. We focused on this population to better understand the current perspectives of teachers at the end of the 2021–2022 school year. Researchers distributed an anonymized link to the survey to personal networks and posted the link on social media sites. The survey data was collected based on the participants’ consent with minimum demographic information. Using social media platforms allowed for an extensive sample of teachers from across the United States to participate in the current study.

### Participants

The collected data included 830 PK-12 teachers representing 49 of the 50 U.S. states; however, if a participant had missing data, they were not included in the final analysis. Participants were 40 years of age (*M* = 40.41, *SD* = 10.38), had almost 13 years of teaching experience (*M* = 13.26, *SD* = 8.97), and were overwhelmingly White (86.8%), female (87.8%), and taught at the elementary level (PK-5; *N* = 339). Just over half of the participants (61.4%) taught in a Title 1 school, and more than four in five (82.8%) taught in a traditional public school. Almost half of the participants (48.1%) taught in a suburban school, about a quarter taught in an urban setting (25.3%), and the rest taught in a rural or small-town setting. The collected data included some missing cases at random with uncompleted surveys (*N* = 6), which were lower than 0.8% of the sample. To increase the accuracy of result interpretation, the moderated mediation model included 824 samples in the final data analysis, excluding the six with incomplete survey data.

### Measurements

The electronic survey included questions about teacher and school demographics, teacher burnout, and school resources (e.g., administrative support within the classroom and technology support, such as 1:1 student devices and learning management systems). The survey included scales of administration support [51], job satisfaction [34], and teacher autonomy [52]. In this study, the constructs also showed statistically acceptable reliability: administrative support (6 items, α = .91), job satisfaction (3 items, α = .86), and teacher burnout (3 items, α = .89). Example questions included “I believe that my efforts in the classroom are unappreciated by the administrators” (administrative support scale), “I enjoy working as a teacher” (job satisfaction scale), and “I feel emotionally drained from my work” (burnout). Previous studies conducted throughout the COVID-19 pandemic indicated that teachers reported increased workloads along with high levels of stress, anxiety, and burnout [e.g., 11–13]. Therefore, the researchers chose to use a brief burnout scale to minimize the time and effort required from teachers at the end of the school year. While the researchers aimed to avoid adding unnecessary burdens, we felt it was essential to assess teacher well-being, particularly given recent reports of increased teacher attrition [53,54]. Also, to explore the moderation effect, we used the mean score of teacher autonomy scales (9 items, α = .85) presented teachers with questions about their perspectives on a 6-point scale agreement, for example, “I control how I use my scheduled classroom time” (teacher autonomy). All constructs included in this study showed acceptable reliability levels higher than 0.85.

### Data analyses

We estimated the moderated mediation analytic model, the regression-based approach that Hayes [55] suggested. The central idea of this analytic model is to explore the mediation effect by combining other possible moderators that may impact the regression paths [56]. The moderated mediation model explores how the mediated effect may differ depending on the moderation conditions [57]. Based on the correlation results among the variables, we tested a simple mediation model with three constructs (i.e., teachers’ perceived administrative support, job satisfaction, and burnout). In the final analytic model, we added two moderators (i.e., teachers’ autonomy and teaching experience) and analyzed the moderated mediation effects. Also, we used the Johnson-Neyman technique to estimate indirect effects by detecting the significant range of confidence intervals [58]. Using the software *SPSS* (*ver*. 28), we reported the results from the bootstrapping method at a level of 10,000 and the moderation effect at different *SD* levels (e.g., at -1 *SD*, mean, +1 *SD* level).

## Results

First, we tested correlations among the five continuous variables (i.e., teachers’ perceived administrative support, job satisfaction, burnout level, the feeling of autonomy, and teaching experiences) regarding RQ1. *Table 1* shows how the variables were correlated with each other. We found that four variables of administrative support, job satisfaction, burnout, and teacher autonomy were significantly correlated. For example, teachers’ perceived administrative support was positively correlated with their job satisfaction (*p* < .001), the feeling of autonomy (*p* < .001), and teaching experience (*p* < .01); on the other hand, it negatively correlated with burnout levels (*p* < .001). Also, teachers’ job satisfaction in the post-COVID era was positively correlated with their feeling of autonomy (*p* < .001) and teaching experiences (*p* < .01); however, it was negatively correlated with teachers’ burnout level (*p* < .001). Teacher burnout showed a significant negative correlation with all other four variables. The results showed that teachers’ feeling of autonomy showed significant positive relationships with administrative support (*p* < .001) and job satisfaction (*p* < .001); on the other hand, there was a negative relationship between autonomy and burnout (*p* < .01). There was no significant relationship between teacher autonomy and their teaching experience (*p* > .05). See *Table 1* for means, standard deviations, and a correlation matrix for these constructs.

**Table 1:** Correlation among the variables (Listwise N = 824).

	*M*	*SD*	1	2	3	4	5
1. Admin Support	21.34	7.51	1				
2. Job Satisfaction	14.33	4.26	.403***	1			
3. Teacher Burnout	15.38	3.27	-.419***	-.584***	1		
4. Teacher autonomy	3.39	0.96	.573***	.394***	-.440***	1	
5. Teaching experience	13.28	8.96	.116**	.123***	-.120**	-.002	1

* *p* < .05

** *p* < .01, and

*** *p* < .001.

Next, we tested a simple mediation model using three variables (i.e., administrative support, job satisfaction, and burnout) with a bootstrapping method (see *Table 2*). Specifically, we explored a direct/indirect effect between perceived administrative support and burnout levels by a mediator of teachers’ job satisfaction. We found that there was both a significant direct effect (*b* = -.095, *CI* [-.121, -.070]) and an indirect effect (*b* = -.087, *BootCI* [-.105, -.070]) between teachers’ perceived administrative support and burnout levels. *Table 2* shows the results of the simple mediation model. The total effect of teachers’ perceived administrative support on burnout was significant (*b* = -.183, *CI* [-.210, -.155]), considering both direct/and indirect effects. Regarding RQ2, the total mediation effect model significantly explained the path regressions among three variables with a good explanation power level (*R* = .419, *R*^2^ *=* .175, *F* (1, 822) = 174.880, *p* < .001).

**Table 2:** A simple mediation model results by mediator teachers’ job satisfaction (N = 824).

	***coefficient B***	***t***	***95CI***
Total effect of Admin support on Burnout	-.184	-13.224***	[-.210, -.155]
Direct effect of Admin support on Burnout	-.095	-7.299***	[-.121, -.070]
	***coefficient B***	***BootSE***	**(*Boot 95 CI*)**
Indirect effect of Admin support on Burnout mediated by Job satisfaction	-.087	.009	(-.105, -.070)

* *p* < .05, ** *p* < .01, and

*** *p* < .001.

Finally, we analyzed a moderated mediation model including two moderating variables of teachers’ autonomy and teaching experience to the previous simple mediation model (Tables 3 through 5), including the multiple moderation effects; the final analytic model showed a different path regression pattern compared to a simple mediation model. Regarding RQs 3 and 4, *Tables 3* and *4* show the final model results for both with and without bootstrapping settings. Based on the statistical structure of the moderated mediation model, we explained the complex regression results with two parts with the potential two outcome variables in our model (i.e., teachers’ job satisfaction and burnout). Regarding the first outcome of teachers’ job satisfaction (see *Table 3*), the overall model showed a good explanation of power level and statistically significant regression results (*R* = .457, *R*^2^ *=* .209, *p* < .001). In this model, teachers’ perceived administrative support and teacher autonomy could not significantly predict job satisfaction level (*p* > .05). Although those two variables were correlated with teachers’ job satisfaction in RQ1, in the multiple regression model considering moderation effect, they were not significant predictors on teachers’ job satisfaction. Interestingly, we found that teachers’ feeling of autonomy moderated the relationship between administrative support on job satisfaction. The interaction effect of teachers’ autonomy between administrative support and teachers’ job satisfaction was statistically significant (*ΔR*^2^ = .007, *F* (1,820) = 7.01, *p* = .008).

**Table 3:** Regression model results with teachers’ job satisfaction (N = 824).

**Outcome** **(Job satisfaction)**	***coefficient B*** **(*BootMean*)**	***SE*** **(*BootSE*)**	***t***	***95CI*** **(*Boot 95CI*)**
Intercept	10.412 (10.380)	1.214 (1.367)	8.574***	[8.028, 12.795] (7.742, 13.103)
Admin support	.002 (.003)	.060 (.065)	.031	[-.116, .119] (-.125, .131)
Teacher autonomy	.154 (.168)	.387 (.435)	.399	[-.605, .913] (-.696, 1.017)
Admin support x Teacher autonomy	.044 (.043)	.012 (.108)	2.647**	[.011, .076] (.007, .079)
**Moderation Effect**	***coefficient B***	***SE***	***t***	***95CI***
Teacher autonomy at -1 *SD*	.108	.027	4.062**	[.056, .161]
Teacher autonomy at mean	.151	.022	6.988***	[.108, .193]
Teacher autonomy at +1 *SD*	.193	.027	7.145***	[.140, .246]

*Note*. In this model, teacher autonomy was set as the moderator.

*p < .05

**p < .01, and

***p < .001.

**Table 4:** Regression model results with teachers’ burnout (N = 824).

Outcome (Burnout)	*coefficient B* (*BootMean*)	*SE* (*BootSE*)	*t*	*95CI* (*Boot 95CI*)
Intercept	15.287 (15.257)	1.127 (.926)	13.565***	[13.075, 17.499] (13.464, 17.116)
Admin support	.073 (.072)	.042 (.038)	1.722⸆	[-.010, .156] (-.002, .146)
Job Satisfaction	.094 (.098)	.082 (.079)	1.149	[-.066, .254] (-.058, .253)
Teacher autonomy	1.617 (1.628)	.313 (.282)	5.160***	[1.002, 2.232] (1.062, 2.173)
Teaching experience	.065 (.064)	.033 (.030)	1.951⸆	[-.000, .130] (.004, .123)
Admin support x Teacher autonomy	-.037 (-.036)	.012 (.012)	-3.037**	[-.060, -.013] (-.060, -.013)
Job satisfaction x Teacher autonomy	-.106 (-.107)	.022 (.022)	-4.834***	[-.149, -.063] (-.151, -.063)
Job satisfaction x Teaching experience	-.005 (-.005)	.002 (-.005)	-2.522*	[-.009, -.001] (-.010, -.001)

⸆ *p* < .10

* *p* < .05

** *p* < .01, and

*** *p* < .001.

Regarding the second outcome of teacher burnout (see *Table 4*), the overall model showed good explanatory power level and statistically significant regression results (*R* = .671, *R*^2^ *=* .451, *p* < .001). In this model, teachers’ perceived administrative support and job satisfaction levels were not directly linked with the teachers’ burnout (*p* > .05); however, we found that all moderation effects were statistically significant and had meaningful interaction effects with other predictors on teachers’ burnout levels. The results of the final analytic model showed that teachers’ feeling of autonomy was a significant moderator in multiple path regressions. In *Table 5*, we also tested the different moderation effects with the pick-a-point approach (i.e., at -1 *SD*, mean, +1 *SD* level). The results showed that teachers with greater autonomy and teaching experiences showed significantly lower burnout levels in the post-COVID era.

**Table 5:** Conditional effects on teachers’ burnout at different values of the moderators.

**Conditional direct effect of admin support on burnout**	***coefficient B***	***SE***	***t***	***95CI***
Teacher autonomy at -1 *SD*	-.016	.018	- .886	[.056, .161]
Teacher autonomy at mean	-.051	.014	-3.554**	[.108, .193]
Teacher autonomy at +1 *SD*	-.086	.019	-4.524***	[.140, .246]
**Conditional indirect effects of** **admin support—job satisfaction—burnout**	***coefficient B***	***Boot*** ***SE***	**(*Boot 95CI*)**
Teacher autonomy at -1 *SD*	Teaching experience at -1 *SD*	-.020	.006	(-.033, -.009)
	Teaching experience at mean	-.025	.007	(-.040, -.012)
	Teaching experience at +1 *SD*	-.030	.009	(-.049, -.014)
Teacher autonomy at mean	Teaching experience at -1 *SD*	-.043	.008	(-.059, -.029)
	Teaching experience at mean	-.051	.008	(-.067, -.035)
	Teaching experience at +1 *SD*	-.058	.010	(-.078, -.039)
Teacher autonomy at +1 *SD*	Teaching experience at -1 *SD*	-.075	.013	(-.103, -.050)
	Teaching experience at mean	-.084	.014	(-.113, -.057)
	Teaching experience at +1 *SD*	-.094	.016	(-.126, -.062)

⸆ *p* < .10

* *p* < .05

** *p* < .01, and

*** *p* < .001.

**Fig 2:**
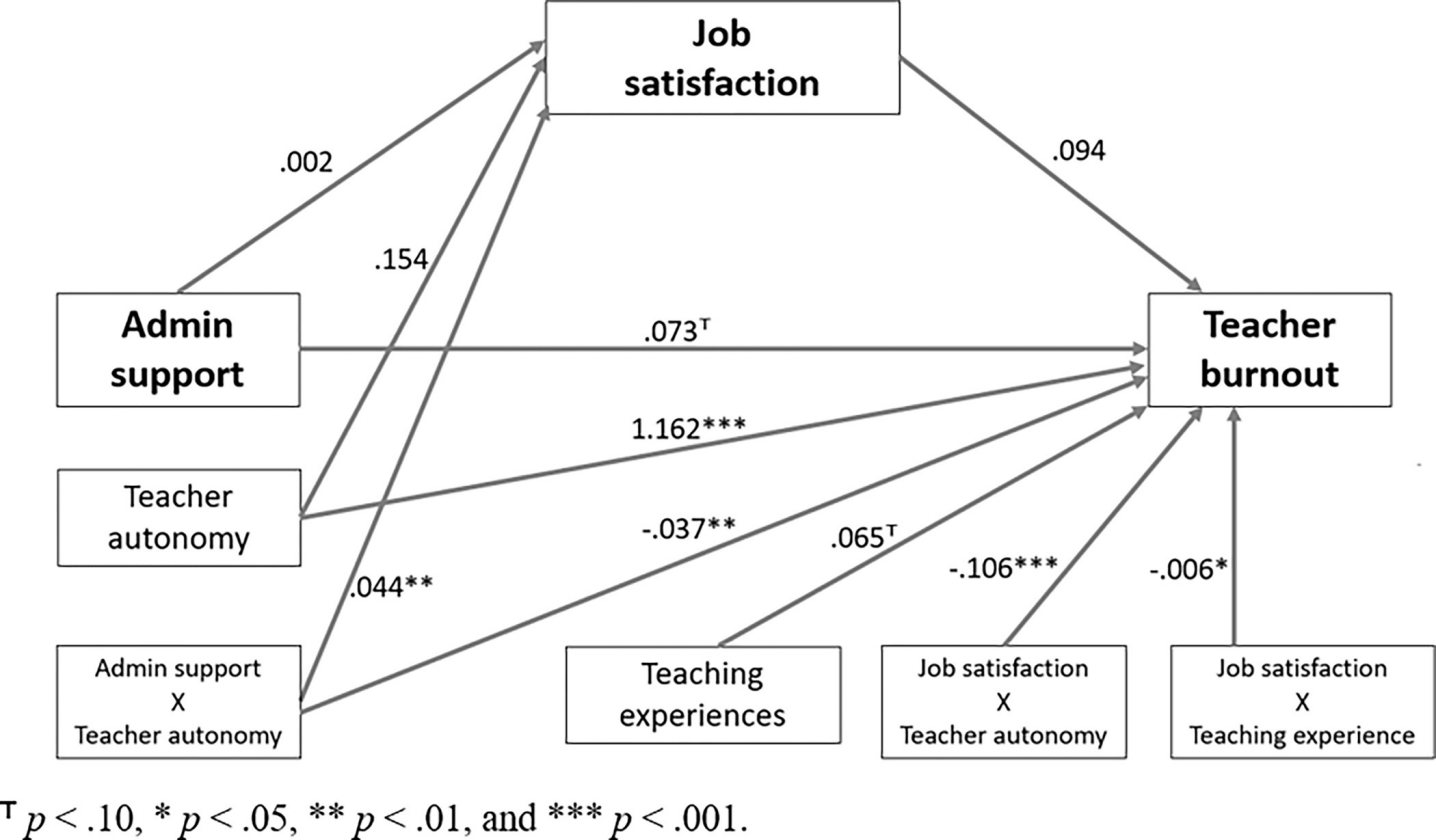
A statistical moderated mediation model of this study.

*Fig 2* shows the results of the interaction effect between a moderator and a predictor in the final moderated mediation model. *Table 6* shows the detailed results of the final analytic model. For example, in a group of teachers with higher autonomy, the linear slopes of administrative support (*ΔR*^2^ = .006, *F* (1,816) = 9.22, *p* = .003) and job satisfaction (*ΔR*^2^ = .016, *F* (1,816) = 23.40, *p* < .001) on teachers’ burnout were significantly greater. In addition, In the final moderated mediation model, we found that teaching experience was a significant moderator between job satisfaction and burnout levels. Specifically, in a group of teachers with longer teaching experiences, the slope of job satisfaction on burnout levels was statistically greater than in teachers with shorter teaching experiences (*ΔR*^2^ = .004, *F* (1,816) = 6.37, *p* = .012).

**Table 6:** Test results of interaction effects between the moderator and predictor (N = 824).

Outcome (Burnout)	*Δ R* ^*2*^	*F*	*df 1*	*df 2*	*p*
Admin support x Teacher autonomy	.006	9.224	1	816	.003**
Job satisfaction x Teacher autonomy	.016	23.400	1	816	< .001***
Job satisfaction x Teaching experience	.004	6.366	1	816	.012*

*Note*. In this model, teacher autonomy and teaching experience were set as moderators.

* *p* < .05

** *p* < .01, and

*** *p* < .001.

## Discussion

The results of this study support the idea that both individual and environmental factors can explain teacher burnout after returning to school from COVID-19 quarantine. With many in-service teachers in the United States still reporting high levels of burnout symptoms or considering leaving the profession after COVID-19 [11,15,28], this study focused on exploring various psychological factors that could provide a deeper understanding and insight into the issue of teacher burnout. These findings add to the literature about teachers’ emotional difficulties in the post-COVID era and can lead to possible suggestions for teachers’ well-being.

First, the correlation results showed that teachers’ burnout level was negatively related to perceived administrative support, job satisfaction, autonomy, and teaching experiences. The current results supported other previous studies’ findings that appropriate administrative support is associated with teachers’ lower stress and burnout levels [3,9,13,17,25]. In this study, we hypothesized that more experienced teachers who were less familiar with technology use would experience higher burnout levels due to the demands of the changed teaching environment during the pandemic; however, the results showed that low job satisfaction and high burnout levels were more prevalent among teachers with fewer years of classroom experience. This study’s results suggest that novice teachers may need additional support to balance their emotional and pedagogical needs as they transition to the teaching career [59]. In the correlation results, all other variables were significantly correlated with a period of teaching experience, excluding only teacher autonomy. Regarding this, we found that teachers’ feelings of autonomy were not related to their teaching experiences (e.g., novice teachers vs expert teachers). Rather than being a direct predictor of teachers’ burnout, feelings of autonomy could explain different teachers’ burnout levels through the various interaction effects with other psychological factors (i.e., perceived administrative support and job satisfaction) [47].

Next, a simple mediation model showed that teachers’ perceived administrative support was directly and indirectly linked to teachers’ burnout. Pearson and Moomaw [16] described teachers’ perceived administrative support associated with various school decision-making processes; when teachers perceive that administrators value teacher opinions in the decision-making process, it can positively affect their work experiences. Also, we found that teachers with lower job satisfaction tended to have greater burnout scores than others with higher job satisfaction. This relationship matters and may cause less satisfied teachers to leave their jobs due to the burden of teaching during the post-COVID-19 period [11]. These results suggest that exploring the various factors that lead to low job satisfaction among teachers and finding appropriate solutions is important in preventing teacher burnout [41,47]. For example, during COVID-19, online learning environments, communications with parents, and anxiety about school safety were pointed out as the major reasons for the burden of teaching jobs [13,45]. However, the current findings show that teacher well-being is still not recovering immediately, even after schools transitioned back to in-person learning and vaccines were readily available long before the current study’s data collection point in 2022. Providing emotional and systematic support for teachers considering their needs related to the post-COVID-19 teaching environment should be further discussed in the PK-12 education system.

Finally, the results of the final model, including the moderator and mediator, highlight the importance of teacher autonomy in navigating the pandemic and recovering teachers’ mental health in schools in a post-pandemic era. The evidence of this study showed that the relationships among the variables (i.e., teachers’ perceived administrative support, job satisfaction, and burnout) might differ by teachers’ feelings of autonomy. For example, although teachers’ autonomy did not directly link to their job satisfaction post-COVID-19, it moderated the relationship between teachers’ perceived administrative support and job satisfaction. In previous studies, researchers noted that supportive school environments and including teachers in school decision-making processes helped reduce anxiety and work-related stress levels [2,16,60]. According to the current study’s results, teacher autonomy was a considerable moderator that made different interaction effects combined with other teachers’ psychological variables (i.e., teacher job satisfaction and burnout). For example, administrative support and teacher autonomy were not solely directly linked to teachers’ job satisfaction; however, teachers with high perceived autonomy and administrative support tended to show higher job satisfaction and lower burnout levels than others. Conclusively, the final model, including the moderator of teachers’ autonomy, provided better explanations of teachers’ burnout prediction in the post-COVID teaching environments.

Schools in the United States have gradually transitioned back to in-person learning, which has led to most schools looking at how they did in pre-COVID-19 educational conditions [6,28]. Even though the environments have returned to normal, it does not equate to teachers and students returning to their pre-pandemic form. It will take time and effort for all teachers and students to adjust to the rapidly changing educational landscape and recover mentally. In this study, we tried to better understand important variables that will help us understand the post-COVID-19 educational landscape. Waters, Cameron [61] suggested using positive psychology approaches to help families, schools, and workplaces transition to a more normal environment. For instance, the implications of negative situations that teachers experienced during the pandemic can bring positive insights into teachers’ mental health recovery. Many teachers saw a reduction in their classroom autonomy during COVID-19, some as a result of following the Centers for Disease Control and Prevention (CDC) guidelines. Teachers often also felt they were excluded from primary administrative decision-making. However, in the post-COVID-19 era, if all stakeholders consider the importance of the teaching environment and thus, supporting teacher autonomy back to normal, it would be the appropriate solution to improve the teacher shortage crisis.

### Implications

To support teacher well-being in a post-pandemic era, policymakers and school leaders will need to make changes to limit teacher burnout. For one, teachers need more autonomy within their classrooms, including decisions related to the curriculum, instruction, and assessment [16]. This will empower teachers to make decisions that they best see fit for their students and may decrease job stress. In addition, school leaders can focus on establishing a supportive school environment that fosters a culture of collaboration or innovation. A successful environment may include dedicated time for teachers to work with each other, such as professional learning communities [62,63]. Beyond the school environment, policymakers need to allow school leaders to make decisions that are best for teachers based on a variety of school circumstances, which can contribute to greater teacher autonomy at the school level. By taking these steps, policymakers and school leaders can create a more supportive environment for teachers, ultimately benefiting students. While the pandemic is in the rearview mirror as of this writing, this study’s findings remain relevant for future challenges in schools that may lead to increased workloads and elevated levels of stress for teachers.

### Limitations and conclusion

The current study provides insight into teacher perspectives at the end of the 2021–2022 school year; however, it includes limitations that future research should consider. As former teachers, we wanted to limit the time required of teachers to participate in this study, and we did not want our survey to substantially add to teacher workloads at the end of the school year. We recognize that research involves tradeoffs. As a result, we opted for a shorter, three-item scale to measure the construct of teacher burnout. Though this was not as robust as more established measures such as the Maslach Burnout Inventory (MBI), many empirical studies conducted in the context of urgent educational needs during the pandemic have utilized short versions of surveys for teachers [e.g., 12,42,64]. Decisions made when conducting research are full of tradeoffs. Although using a 3-item burnout measure may lead to construct validity issues, it asked less of teachers, many of whom were already experiencing elevated work demands. Future studies can consider more robust measures of burnout, but we urge researchers to continue to consider the time requested of teachers.

The current research is a cross-sectional study that only provides a snapshot of teacher perceptions and feelings. Therefore, more longitudinal studies may be necessary to understand how teacher perceptions and feelings change over time. The current study sampled teachers through social media through an anonymous link to reach a large number of teachers. It is possible that teachers who engage more regularly with social media applications differ in important ways from those who do not or those who did not encounter our invitations to participate in this research. Future research should focus on a more randomized selection through multiple data collection resources of teachers to include diverse populations and increase the generalizability of the findings. Future research could explore additional questions, such as the following. While this study included a sample large enough for single-group analysis, the participant group, similar to the general demographic profile of U.S. PK-5 teachers, was predominantly composed of White female teachers. To adequately examine results that may vary across different teacher demographics, future studies have to focus on collecting larger samples within each subgroup. Furthermore, we recommend advancing latent profile and latent transition analyses in future studies by using data from these diverse samples to create potential subgroups that incorporate social and cultural contexts beyond the distinguishable demographic variables.

Overall, evidence from this study suggests that supporting teachers psychologically and administratively is important to return to normal schooling for both students and teachers. These findings are key as teacher attrition continues to grow across the United States, and school leaders should look to take action to support teachers’ well-being. School leaders can take actionable steps to limit teacher burnout by supporting teachers within the school environment. Reflecting on teachers’ voices and needs in PK-12 teaching environments and providing appropriate political and emotional support for teachers may help them feel satisfied with their work and promote teachers’ mental health.
